# Species Interactions during Diversification and Community Assembly in an Island Radiation of Shrews

**DOI:** 10.1371/journal.pone.0021885

**Published:** 2011-07-07

**Authors:** Jacob A. Esselstyn, Sean P. Maher, Rafe M. Brown

**Affiliations:** 1 Biology Department, McMaster University, Hamilton, Ontario, Canada; 2 Odum School of Ecology, University of Georgia, Athens, Georgia, United States of America; 3 Biodiversity Institute and Department of Ecology and Evolutionary Biology, University of Kansas, Lawrence, Kansas, United States of America; University of Western Ontario, Canada

## Abstract

**Background:**

Closely related, ecologically similar species often have adjacent distributions, suggesting competitive exclusion may contribute to the structure of some natural communities. In systems such as island archipelagos, where speciation is often tightly associated with dispersal over oceanic barriers, competitive exclusion may prevent population establishment following inter-island dispersal and subsequent cladogenesis.

**Methodology/Principal Findings:**

Using a combination of tools, we test the hypothesis that the distributions of shrew (*Crocidura*) species in the Philippines are the result of competitive exclusion preventing secondary invasion of occupied islands. We first compare ecological niche models between two widespread, allopatric species and find statistical support for their ecological similarity, implying that competition for habitat between these species is possible. We then examine dispersion patterns among sympatric species and find some signal for overdispersion of body size, but not for phylogenetic branch length. Finally, we simulate the process of inter-island colonization under a stochastic model of dispersal lacking ecological forces. Results are dependent on the geographic scope and colonization probability employed. However, some combinations suggest that the number of inter-island dispersal events necessary to populate the archipelago may be much higher than the minimum number of colonization events necessary to explain current estimates of species richness and phylogenetic relationships. If our model is appropriate, these results imply that alternative factors, such as competitive exclusion, may have influenced the process of inter-island colonization and subsequent cladogenesis.

**Conclusions/Significance:**

We interpret the combined results as providing tenuous evidence that similarity in body size may prevent co-occurrence in Philippine shrews and that competitive exclusion among ecologically similar species, rather than an inability to disperse among islands, may have limited diversification in this group, and, possibly other clades endemic to island archipelagos.

## Introduction

Theory predicts that closely related species cannot coexist until they have diverged sufficiently in ecologically important traits [1]–[5]. Ecological differentiation may occur rapidly in clades undergoing adaptive radiation [6]–[8], but much of biological diversity probably results from speciation across geographic barriers, with relatively little attendant divergence in ecologically important traits [9]–[13]. In many cases, ecological diversification may happen early in a clade's history, with later events producing ecologically similar species [14], [15]. If many species are indeed generated without producing significant ecological differences, competition may result when closely related, initially isolated species come into contact, and these interspecific interactions may result in competitive exclusion, thereby preventing allopatric cladogenesis.

Such coevolutionary thinking was endorsed enthusiastically until the 1970s [2], [16], but subsequently has been treated with caution [17]–[21]. Nevertheless, studies continue to document patterns consistent with the notion that competition plays a role in community assembly [22]–[27]. Although most authors acknowledge some role of competition in shaping communities under particular circumstances [e.g., 20], many questions remain as to competition's potency, pervasiveness, results, and detectability [17], [18], [28]. In adaptive radiations, competition is often viewed as a factor promoting species diversification [6], [29]. However, in radiations that diversify primarily across geographic barriers with little change in ecologically important traits, competition has the potential to prevent speciation, by limiting the ability of individual species to expand over barriers into the range of other, closely related species.

Unfortunately, competition is difficult to either document or refute in empirical studies of free-living organisms. Much of the argument for competitive exclusion therefore derives from theoretical treatments [30], empirical microcosm studies [24], [31], [32], and correlational studies of patterns of species co-occurrence in natural communities [25], [33], [34]. Thus, the pervasive observation of ecologically similar sister species with abutting peripatric, or narrowly overlapping parapatric distributions, stands as one of the most often-cited forms of evidence for competitive exclusion [e.g., 9], [35]. However, other processes, such as vicariant isolation, may generate the same pattern [17], [36], making it difficult to distinguish among potential underlying mechanisms. Nevertheless, if competition for habitat results in exclusion and is the cause of a particular pair of abutting ranges, then the competing species *must* occupy similar ecological space. Until very recently, techniques for quantifying ecological similarity were limited, and primarily anecdotal [36]. However, with the advent of ecological niche modeling and associated statistical tests, an objective, coarse-resolution means of assessing ecological similarity is now available [37]–[39].

Most discussion of niche evolution centers on the Grinnellian model [e.g., 11], [15], which emphasizes the environmental dimensions occupied by a species. This conception is useful from a practical standpoint because of the availability of environmental data, and we focus on it here. If Grinnellian niches are conserved over evolutionary time scales [11], [15] and niche similarity results in competition, then secondary colonization of habitats occupied by closely related species should lead to either extirpation of one species (exclusion) or character displacement in some ecologically significant character that lessens competition and permits coexistence [e.g., 28], [33]. If so, then within clades that primarily undergo speciation across geographic barriers, co-occurring species are expected, on average, to be more distantly related and/or more different ecologically from one another than expected under a model of random draws from the regional species pool. In other words, if competition plays a role in determining the outcome of inter-island dispersal events (i.e., establishment vs. failure to colonize), sympatric species should be overdispersed (more dissimilar than expected by chance) on the phylogeny and/or in traits that result in ecological differences between species [25], [34], [40].

Here, we combine a variety of approaches to explore the potential role of competitive exclusion in limiting inter-island colonization, and hence speciation, in a group of shrews (genus *Crocidura*) endemic to the Philippine archipelago. Shrews are widely distributed in the Philippines, occurring on nearly all islands that have been adequately surveyed for small terrestrial mammals [41]. Most islands have a single species of *Crocidura* on them, implying that ecological interactions may prevent coexistence among these closely related species.

To determine whether Philippine *Crocidura* could potentially compete with one another for habitat, we employ ecological niche modeling to assess crude similarity of potential habitat use. We then test for overdispersion of sympatric species in terms of their phylogenetic relatedness and body size, with the goal of understanding whether the few cases of co-occurrence are non-random. Body size represents an important ecological trait that may influence the ability of species to coexist [42]–[44], potentially due to its relationship with other factors, such as metabolic and reproductive rates, prey preferences, and vulnerability to predators [45]. As a final treatment of questions related to the potential role of species interactions in determining current species richness, we simulated the process of inter-island colonization to determine whether the current distribution of *Crocidura* could be generated with a random model of dispersal that lacks ecological forces.

### Geographic Setting

The Philippines has a remarkably complex geological history, in which a combination of volcanic activity, subduction, and island accretion altered the distribution of land dramatically over the history of the archipelago (approximately the last 30 My) [46]–[48]. Geological history and its effect on biological diversity in the Philippines have been discussed extensively in several papers [49]–[55], and references cited therein.

**Figure 1:**
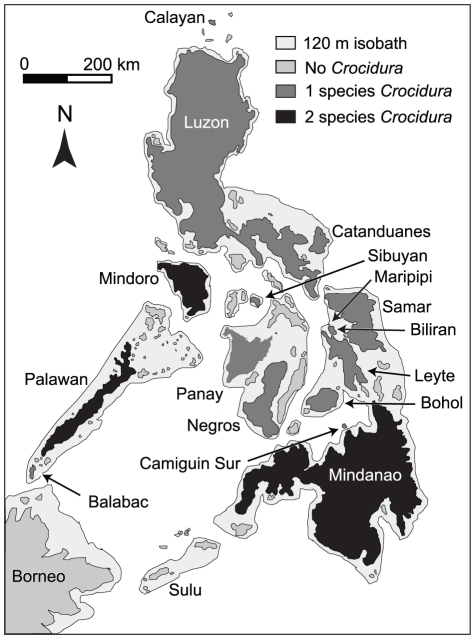
Map of the Philippines. The extent of land during Pleistocene sea-level low-stands corresponding to the 120 m isobath is shown in light gray. Modern islands are shaded according to their shrew diversity, with islands lacking *Crocidura* records as medium gray, islands with one species of *Crocidura* as dark gray, and islands with two species as black (Borneo excluded). Species recorded from each island are given in Table 1.

However, with regard to the relatively recent ecological and evolutionary processes considered here, the most relevant aspect of the geographic history of the archipelago is that of sea-level fluctuations, and the resulting aggregation of islands currently separated by shallow seas. Because the large complex islands of the Philippines are the product of accretion, rather than breakup, of paleoislands [46]–[48], geologically driven vicariance is largely absent from the Philippines; all speciation events in Philippine *Crocidura* are thus thought to be the result of inter-island colonization [41]. However, sea levels fluctuated widely from the late Pliocene through the Pleistocene, and resulted in cycles of connection and isolation among modern islands in the Philippines [56]–[61]. When sea levels were low (−120 m) six major islands were formed, here termed greater Luzon, Mindanao, Mindoro, Negros–Panay, Palawan, and Sulu (Fig. 1). We refer to these as Pleistocene Aggregate Island Complexes (PAICs) [62]. Although Plio-Pleistocene sea-level fluctuations varied in duration, magnitude, and local effect, islands separated by channels currently at least 140 m deep have probably remained isolated throughout their history [55]. Presumably, the repeated connections among neighboring islands allowed for dispersal of plants and animals between modern islands within PAICs. Phylogeographic and taxonomic evidence suggests the effect is important, but not universal [50], [52], [55], [63]–[67]. Because of the absence of geologically driven vicariance in the archipelago [46]–[48], we consider the history of sea-level fluctuations more important than tectonic processes to understanding the colonization history of Philippine shrews. Given the lack of tectonic vicariance, all currently isolated shrew populations must be the result of over-water colonization, or colonization over land bridges.

### Distributional Patterns of Shrews in the Philippines

*Crocidura* shrews are widely distributed in the Philippines; they have been documented on all but one of the PAICs (Sulu) and on a few small oceanic islands (Fig. 1; Table 1) [41], [49], [68], [69]. One species, *C. tanakae*, occurs only at the northern extremity of the Philippines, in the Batanes Islands—it is closely related to populations from Taiwan and the Asian mainland, and a distant relative of other Philippine *Crocidura* [70]; as it is part of a distinct biogeographic setting and species pool, we exclude it from further consideration. Among the remaining nine species, at least seven are members of an endemic Philippine clade that occurs throughout the country, from Calayan in the north, to Palawan, Balabac, and Mindanao in the south (Fig. 1; Table 1) [41]. One species (*C. grandis*) has not been recorded in over a century and is known only from the holotype [71], but likely is a member of the endemic Philippine clade [68]. Another species (*C. batakorum*), occurs on Palawan and is most closely related to an endemic Sulawesian radiation of *Crocidura* [41]. Among the nine species we consider here, most are endemic to a single PAIC or oceanic island (Fig. 1; Table 1). The two exceptions are *C. grayi*, which occurs on Greater Luzon, and on Mindoro and Calayan islands, both of which are isolated by deep water. The other is *C. beatus*, which occurs on the islands of Greater Mindanao, but also on Camiguin Sur, a small, young volcanic island that has remained isolated throughout its existence [72]. Thus, most islands in the Philippines hold single species of *Crocidura*, but two species are found on the islands of Mindanao (*C. beatus* and *C. grandis*), Mindoro (*C. grayi* and *C. mindorus*), and Palawan (*C. batakorum* and *C. palawanensis*: Fig. 1; Table 1). The taxonomy of Philippine *Crocidura* has recently been revised and is relatively well understood [41], [68], [69], [73].

**Table 1:** Distribution of shrews (*Crocidura*) in the Philippines (excluding the Batanes Islands).

Species	Island	Area (km^2^)	Pleistocene Island	GenBank Accessions: CytB/ND2
*Crocidura batakorum*	Palawan	11,785	Palawan	FJ813976/FJ814541
*Crocidura beatus*	Biliran	498	Mindanao	
	Bohol	3864	Mindanao	
	Camiguin Sur	249	–	FJ813985/FJ814550
	Leyte	7213	Mindanao	
	Maripipi	22	Mindanao	
	Mindanao	96,467	Mindanao	FJ813844/FJ814410
	Samar	13,429	Mindanao	
*Crocidura grandis*	Mindanao	96,467	Mindanao	
*Crocidura grayi*	Calayan	196	–	FJ813930/FJ814495
	Catanduanes	1513	Luzon	
	Luzon	107,170	Luzon	FJ813850/FJ814416
	Mindoro	9735	Mindoro	FJ813932/FJ814497
*Crocidura mindorus*	Mindoro	9735	Mindoro	FJ813840/FJ814406
*Crocidura negrina*	Negros	13,670	Negros–Panay	FJ813957/FJ814522
*Crocidura ninoyi*	Sibuyan	449	–	FJ813841/FJ814407
*Crocidura palawanensis*	Balabac	306	Palawan	
	Palawan	11,785	Palawan	FJ813978/FJ814543
*Crocidura panayensis*	Panay	12,300	Negros–Panay	FJ813945/FJ814509

The Pleistocene Island column indicates to which Pleistocene Aggregate Island Complex the island belongs, if any. GenBank accession numbers are given for populations included in the test of phylogenetic dispersion.

Herein, we treat populations on islands separated by deep ocean channels, which have never been connected to one another [58], [59], as species. We adopt this strategy because taxonomy is conservative in its recognition of recently diverged species, requiring diagnostic characters, which are unlikely to be present in the youngest species. Furthermore, we think gene flow between populations on permanently isolated islands is probably very rare, and populations on these islands should therefore be treated as species in analyses of evolutionary processes. This approach is consistent with Wiley's evolutionary species concept [74].

## Methods

### Ethics Statement

Permits to collect scientific specimens were provided by the Protected Areas and Wildlife Bureau of the Philippine Department of Natural Resources. Field protocols were approved by the University of Kansas IACUC #158-02.

### Modeling Potentially Suitable Ecological Space

Most species of Philippine *Crocidura* are known from only a few localities. Two species (*C. grayi* and *C. beatus*), however, have moderately wide geographic distributions, each with numerous spatially unique, vouchered localities [66]. To characterize ecological niches of Philippine *Crocidura*, we used all known sampling localities to generate ecological niche models (ENMs) for *C. grayi* from Greater Luzon and *C. beatus* from Greater Mindanao using Maxent 3.3.3 [75].

Maxent uses an algorithm based on the principle of maximum entropy. The result of the algorithm is a probability distribution from the environmental and occurrence data in which the best explanation is that which shows the broadest probability distribution. Maxent fits this distribution subject to particular constraints, in this case, environmental values associated with collection localities. The logistic output is considered by some as an analogue of the probability of species occurrence in a Bayesian context [76]. To convert the resulting map of continuous probabilities to a predicted presence/absence map, we used the lowest probability in our training occurrence data as a threshold, where lower probabilities were considered absence [77].

We generated ENMs using 44 (*Crocidura grayi*) and 33 vouchered localities (*C. beatus*) and raster GIS layers summarizing climate parameters. Climate data consisted of seven WorldClim layers [78] that represent variation in precipitation and temperature (annual mean temperature, mean diurnal temperature range, maximum temperature of warmest month, minimum temperature of coldest month, annual precipitation, precipitation of wettest month, and precipitation of driest month), and are generally uncorrelated with one another [79].

We plotted occurrence points and regions that could be reasonably assumed to have been available for colonization by the species, as “M” in the “BAM” framework [80], [81]. The BAM concept is best visualized as a Venn diagram, in which an organism's geographic distribution is represented by the intersection of the biotic (B), abiotic (A), and movement (M) components of the organism's niche and history [e.g., 82], [83]. M is intended to represent areas the species has explored during its history. For the purpose of this study, M was defined as all islands within the PAIC on which the species occurs. Thus, for *C. grayi* (excluding Mindoro and Calayan populations), this area was represented by Greater Luzon and for *C. beatus* (excluding Camiguin Sur population) it consisted of Greater Mindanao (Fig. 1).

We generated ENMs for each species with current climate data, drawn from their respective M areas. These ENMs were then projected onto the entire Philippine archipelago and northern Borneo using current climate data and Pleistocene reconstructions of environmental layers representing the last glacial maximum (LGM, 20 Kya) [84] and last interglacial (LIG, 135 Kya). We applied the threshold rule derived from the current climate models to each of the climate reconstructions.

As a test of the hypothesis that *Crocidura beatus* and *C. grayi* are ecologically similar, and therefore potential competitors, we calculated the niche overlap metrics, Hellinger's based I and Schoener's D, for the thresholded ENMs projected onto the current, LGM, and LIG climate regimes. Completing these tests over three distinct climate scenarios provides an indication of how consistent any similarities or differences in ecological niches might be, given Pleistocene levels of climate variation. Niche similarity was evaluated using a variant of the background similarity test [39], as implemented in ENM Tools. To produce null distributions of overlap metrics, we generated random occurrence points (44 for *C. grayi* and 33 for *C. beatus*) within the area of M for one of the two species. ENMs were generated in Maxent 3.3.3 from these points within the respective M, projected onto the climate space of the entire archipelago (as above), thresholded with the minimum presence value, and compared to the empirical, thresholded ENM of the other species to calculate the overlap metrics I and D. Nine-hundred-ninety-nine randomizations were completed and projected onto each climate regime. We placed observed overlap values in the resulting null distributions of I and D and calculated one-tailed *P*-values.

### Testing for Phylogenetic Overdispersion

We used previously published mitochondrial DNA sequence data to infer an ultrametric tree for eight of the nine species (*Crocidura grandis* is unavailable) of Philippine *Crocidura* recognized by taxonomy [41], [73], plus all known populations from oceanic islands. Nuclear sequence data were not used as divergences among these species are mostly recent and involve limited differences in available nuclear loci [70]. A concatenated character matrix of Cytochrome b and NADH dehydrogenase subunit 2 (ND2) was used (2184 nucleotides). The matrix is nearly complete, with only 10 characters missing from the 3′ end of ND2 in *C. mindorus*. We included a single individual of each taxonomically defined species, from each of the PAICs and/or oceanic islands on which it occurs. Thus, for the eight taxonomically defined species sampled, a total of 11 individuals (evolutionary species) were included (Table 1), comprising three representatives of *C. grayi* (one each from the islands of Luzon, Mindoro, and Calayan), two of *C. beatus* (one each from the islands of Mindanao and Camiguin Sur), and one of each of the remaining species. Phylogenetic topology and branch lengths were inferred in a Bayesian framework using BEAST v1.5.3 [85]. Six independent runs of 5 million generations were completed using a GTR + Γ model of sequence evolution and Yule speciation prior. Parameters were sampled every 2000 generations and the initial 300,000 generations of each run were discarded as burn-in, leaving 15,000 trees in the posterior distribution. To evaluate convergence among MCMC analyses, trends and distributions of parameters, including the likelihood score, were examined in Tracer v1.4 [86]. The posterior distribution of trees was summarized on a maximum clade credibility tree with branch lengths presented as median heights.

Pairwise patristic distances (i.e., sums of branch lengths separating two terminals) were calculated between all terminals using the DendroPy phylogenetic library [87]. We then calculated a metric of phylogenetic dispersion as



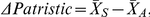



where 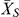 is the mean of pairwise patristic distances separating sympatric species and 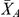 is the mean of pairwise patristic distances separating allopatric species. If Δ*Patristic* is positive, sympatric species are distant relatives, indicating either allopatric speciation resulting from inter-island colonization or, if the value is higher, perhaps the presence of a sympatry threshold and competitive exclusion. If Δ*Patristic* is negative, this indicates either habitat filtering, in which closely related species tend to occur sympatrically because they have similar ecological needs, or within-island speciation. Because no tissue samples of *C. grandis* are available, this test incorporated only two sympatric species pairs (*C. grayi* and *C. mindorus* from Mindoro and *C. batakorum* and *C. palawanensis* from Palawan). To measure significance, we recalculated Δ*Patristic* 2000 times on the empirical matrix of distances, with sympatry (two species pairs) randomized among the terminals, and calculated a one-tailed *P*-value from this distribution. This approach is similar to the widely used Net Relatedness Index (NRI) [40], [88], but allows us to calculate a single measure of dispersion for the regional community and provides an alternative means of testing dispersion patterns where diversity of individual island communities is too low to use the more standard NRI. This test was completed on the maximum clade credibility tree with branch lengths summarized as medians, and on a sample of 600 trees (the last 100 samples from each run) drawn from the posterior distribution.

### Testing for Overdispersion in Body Size

Body size represents an important ecomorphological trait in shrews and many other vertebrates [4], [5], [42], [43], [89], [90]; communities of sympatric species of shrews are often noted for their highly regular distributions of body size [44]. Here, we use the length of the skull as a proxy for body size because it is available from all island populations and can be consistently measured [e.g., 4], [89], [91]. We measured the greatest length of skulls from the posterior margin of the occipital condyles to the anterior margin of the incisors (condylo-incisive length), using digital calipers precise to the nearest 0.01 mm. Only adult specimens, as judged by complete fusion between the basioccipital and basisphenoid bones and fully erupted molars [e.g., 92], were measured (Appendix S1). The average skull length was calculated for each species, and pairwise differences in mean skull length were calculated between all species. As with the phylogenetic dispersion analysis, we included representatives of each taxonomically defined species from all permanently isolated islands on which it occurs. Thus, all of the island populations included in the phylogenetic analysis are represented here. In addition, we include the holotype of *Crocidura grandis*, resulting in the representation of all known species of *Crocidura* from our focal area and inclusion of all three sympatric species pairs (*C. palawanensis* and *C. batakorum* from Palawan, *C. grandis* and *C. beatus* from Mindanao, and *C. grayi* and *C. mindorus* from Mindoro). The test statistic for body-size dispersion was calculated as



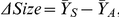



where 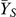 is the mean of differences in body size among sympatric species pairs and 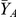 is the mean of differences in body size among allopatric species pairs. A null distribution was generated by randomizing sympatry (three pairs) among the species and recalculating Δ*Size* 2000 times. We then calculated a one-tailed *P*-value by placing the observed value in this distribution. We repeated this exercise using median values of body-size differences to avoid any potentially undue influence of a single value.

Because body size overdispersion could result from either competitive exclusion or character displacement, we also tested for phylogenetic signal in body size using Pagel's lambda [93]. Likelihood scores for untransformed and transformed trees were calculated in R 2.10.1 [94] using the package GEIGER [95] and significance was evaluated with a likelihood ratio test. The result was compared to a chi-square distribution with one degree of freedom.

### Simulating the Process of Island Colonization

We simulated the process of island colonization to determine whether a random dispersal process lacking ecological interactions could generate the known geographic distribution of Philippine *Crocidura*. In other words, we asked whether competitive exclusion might have caused the failure of past inter-island dispersal events after arrival of potential propagules on an occupied island. In doing so, we assumed that all colonization events lead to speciation, which we consider reasonable given our understanding of shrews' limited ability to regularly cross marine barriers and the understanding that all known Philippine shrew species are probably the result of inter-island colonization [41].

A single island was randomly selected as the first island with a shrew population. This seeding event was not counted as a colonization event. From there, colonization events occurred one at a time with the source population selected at random from among occupied islands. The recipient island was selected among all the islands (excluding the source) with a probability derived from the distance between it and the source. The simulations were run with two distinct probability distributions: (1) the probability of colonizing a particular island was inversely proportional to its minimum inter-shore distance from the source island, and (2) this probability was the inverse of the distance squared. We adopted the second approach to account for our expectation that long-distance colonization by shrews should be much rarer than short-distance colonization; squaring the distance results in much lower probability for long-distance colonization. This expectation is based on shrew's fast metabolic rate and small body size, which presumably make it difficult for them to survive long periods of time at sea. The simulated colonization process was repeated until a given number of islands had been colonized. For these simulations, we treated island groups united during Pleistocene sea-level low-stands as single islands. Minimum distances among these PAICs were measured using Google Earth and were taken between the shores of the nearest modern islands with an area ≥100 km^2^ within each PAIC. Simulation code was written in Python and is available at: http://github.com/jesselstyn/Island-colonization.

Because uncertainty exists as to exactly how many islands have extant populations of *Crocidura*, we adopted three geographic scopes in these simulations, including scenarios where 8 of 14 islands, 8 of 9 islands, and 5 of 6 islands must be colonized before the simulation is terminated. The 14-island scenario included all five PAICs and the three oceanic islands with shrew records (Camiguin Sur, Calayan, and Sibuyan; Fig. 1), plus the one PAIC (Sulu) and five oceanic islands lacking a shrew record. The oceanic islands included here are those with an area ≥100 km^2^ and records of at least three native mammal species [96, 97; Oliveros and Esselstyn, unpubl. data]. Thus, we included oceanic islands that are both sufficiently large to support shrew populations (see below) and have been the subject of at least cursory biodiversity inventories (i.e., Babuyan Claro, Camiguin Norte, Lubang, Siquijor, and Tablas islands). In the 9-island scenario, all five PAICs and three oceanic islands with shrew records, plus the largest PAIC lacking a shrew record (Sulu) were included. In the 6-island scenario, only the PAICs were included, leaving out all oceanic islands. We adopted this final approach with the hope of avoiding the uncertainty in shrew presence/absence on small islands.

Under each scenario, the total number of colonization events (speciation events) that had occurred by the time the termination criterion was met was recorded during each of 10,000 replicates. A subset of these replicates, which originated from the most plausible routes of colonization from the continent into the Philippines (Palawan and Mindanao islands), were examined as well, to determine whether the seeded island impacts the number of colonization events necessary for an organism to spread across the archipelago. We then calculated one-tailed *P*-values with these distributions by treating the minimum number of colonization events necessary to generate the known distribution of shrews as the observed value.

In general, large PAICs have been the subject of more intensive biodiversity surveys than smaller islands, and knowledge of their shrew faunas is more complete. Given this bias in survey effort, we decided to limit the simulations geographically to the PAICs, oceanic islands known to have shrew populations, and the larger oceanic islands (≥100 km^2^) with records of at least three native mammal species. By excluding small islands, we assumed there is a lower limit on the area of an island necessary to support a shrew population over evolutionary time scales [e.g., 98]. Among the islands in the area considered here that are known to have shrew populations, Maripipi is the smallest (22 km^2^). However, it was united repeatedly with the larger islands of Greater Mindanao during the Pleistocene, perhaps indicating that it does not provide a meaningful indication of the smallest suitable island. Calayan (196 km^2^) is the smallest island never to have been connected to another island that is known to have a population of *Crocidura*. However, outside the area of our geographic focus, populations of *Crocidura* are found on Batan (35 km^2^) and Sabtang (41 km^2^) islands [68], [70], which were connected to each other, but are isolated from other islands and the continent by deep water. Thus, there is little evidence of shrews being capable of long-term persistence on islands smaller than about 100 km^2^, and we treated this as the minimum area necessary.

## Results

### Modeling Potentially Suitable Ecological Space

**Figure 2:**
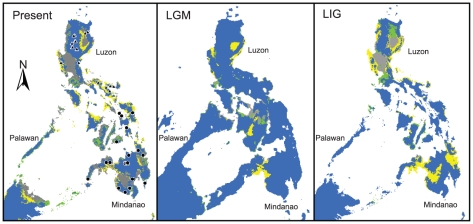
Results of ecological niche modeling. Collection localities used to generate ecological niche models are represented by black triangles (*Crocidura grayi*) and circles (*C. beatus*). Potentially suitable climatic space is shown for *C. beatus* only (green), *C. grayi* only (yellow), and both species (blue) in the Philippines and northern Borneo. Areas identified as unsuitable for both species are shown in gray. Predicted potential distributions are shown for the present, Last Glacial Maximum (LGM), and Last Interglacial (LIG).

Ecological niche models estimate broad geographic overlap in the potentially suitable ecological spaces for *Crocidura beatus* and *C. grayi* (Fig. 2). Both species are predicted to find suitable climatic space across much of the Philippines and northern Borneo under current, LGM, and LIG conditions. Tests of niche overlap failed to reject the null hypothesis that *C. beatus* and *C. grayi* have similar niches, using both metrics of similarity and with independent randomizations of each species' occurrence data, under each climate regime (Table 2). *P*-values were in fact very high, and under a two-tailed approach revealed statistically significant similarity between *C. beatus* and *C. grayi* in five of the 12 tests, with marginal support for similarity in another five tests (Table 2).

**Table 2:** Results of background similarity tests of the predicted ecological niches of *Crocidura beatus* from Greater Mindanao and *C. grayi* from Greater Luzon.

Similarity Metric	Time Period	Empirical Values	*P*-values: *C. grayi* localities randomized	*P*-values: *C. beatus* localities randomized
Hellinger's based I	Present	0.942234	0.992	0.976
	LGM	0.952043	0.991	0.920
	LIG	0.943076	0.959	0.710
Schoener's D	Present	0.990111	0.987	0.961
	LGM	0.989482	0.979	0.930
	LIG	0.993111	0.940	0.634

Ecological niche models used climate data for the present, Last Glacial Maximum (LGM), and Last Interglacial (LIG). *P*-values revealing statistically significant similarity are emphasized with a bold typeface.

### Phylogenetic Dispersion

**Figure 3:**
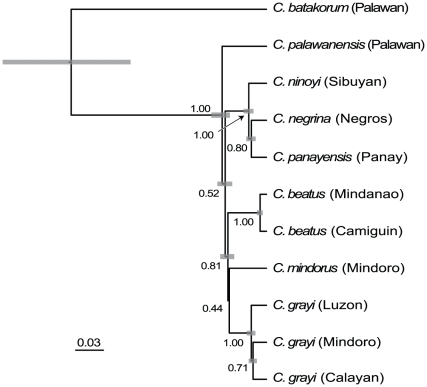
Maximum clade credibility tree for Philippine shrews (*Crocidura*). Terminals are labeled with species names, followed by island names in parentheses. Numbers at internal nodes are posterior probabilities. Gray bars at nodes represent 95% highest posterior densities of node ages on an arbitrary time scale.

**Figure 4:**
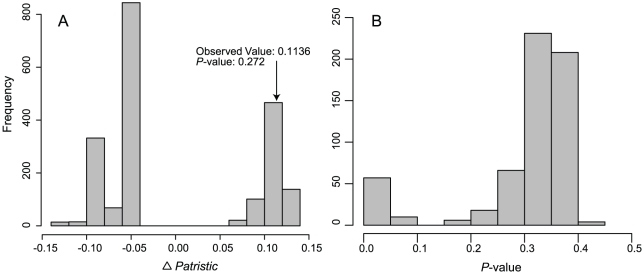
Relatedness of sympatric and allopatric shrews. Panel A shows the distribution of 2000 randomizations of Δ*Patristic* (difference in mean patristic distances between sympatric species pairs and between allopatric species pairs) among species of Philippine *Crocidura*. The observed value and one-tailed *p*-value are indicated. Panel B shows one-tailed *p*-values for Δ*Patristic* from a sample of 600 trees drawn from the posterior distribution.

Phylogenetic inferences were consistent across six independent Markov Chain Monte Carlo analyses. Examination of trends in log-likelihood scores and other parameters suggest that all six runs converged within the first 300,000 generations. Effective sample sizes for all parameters were >200, with most exceeding 1000. The topology inferred here (Fig. 3) is similar to previous estimates [41], differing only in the placement of *Crocidura mindorus*. The phylogenetic position of this species consistently receives low support [41], [66], [70], probably a result of short internal branches. However, as our test is based on branch lengths, the topology is only critical to the extent it affects branch lengths. The test statistic, Δ*Patristic*, was positive, and hence in the direction of overdispersion (Fig. 4); however, its deviation from zero on the maximum clade credibility tree was not statistically significant (*P* = 0.272). All 600 samples we tested from the posterior also had positive values of Δ*Patristic*, but most were not statistically significant (Fig. 4).

### Body-Size Dispersion

**Figure 5:**
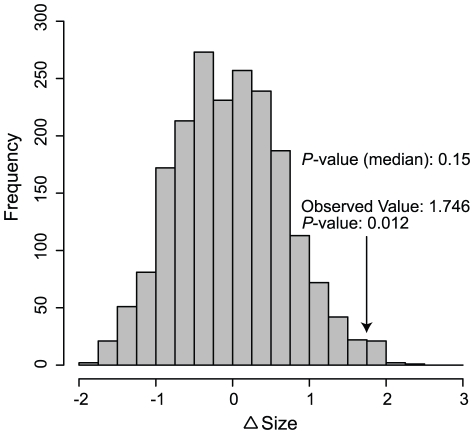
Body size differences between sympatric and allopatric shrews. The distribution of 2000 randomizations of Δ*Size* (difference in the mean difference in skull length between sympatric species pairs and between allopatric species pairs) among species of Philippine *Crocidura* is shown. The observed value and one-tailed *p*-value are indicated, as is the *p*-value when we repeated this analysis using medians, rather than means.

Body sizes, as indexed by average skull length, range from 18.01 to 23.70 mm (Table 3). The empirical value of Δ*Size* (1.746) was greater than the corresponding values from nearly all randomizations (Fig. 5; *P* = 0.012), suggesting that body size is significantly overdispersed in sympatric species pairs of shrews in the Philippines. However, when we repeated this analysis using medians, rather than means, the effect was reduced, with Δ*Size* equaling 1.10 (*P* = 0.155). Phylogenetic signal in body size was marginal (*P* = 0.076).

**Table 3:** Mean condylo-incisive lengths (mm), with standard errors (SE) and sample sizes (*N*) for Philippine species of *Crocidura*.

Species	Island	Mean condylo-incisive length ± SE (*N*)
*C. batakorum*	Palawan	18.01±0.091 (5)
*C. beatus*	Greater Mindanao	20.99±0.143 (13)
*C. beatus*	Camiguin	20.80±0.136 (6)
*C. grandis*	Mindanao	23.70±NA (1)
*C. grayi*	Luzon	20.12±0.091 (23)
*C. grayi*	Calayan	21.17±0.170 (4)
*C. grayi*	Mindoro	19.63±0.032 (15)
*C. mindorus*	Mindoro	22.28±0.141 (4)
*C. negrina*	Negros	22.93±0.215 (8)
*C. palawanensis*	Greater Palawan	23.62±0.145 (27)
*C. panayensis*	Panay	21.45±0.279 (7)
*C*. *ninoyi*	Sibuyan	22.60±0.335 (3)

Measurements were taken from adult voucher specimens collected on Pleistocene islands and oceanic islands (Appendix S1). These lengths were used as a proxy for body size.

### Island Colonization Process

**Figure 6:**
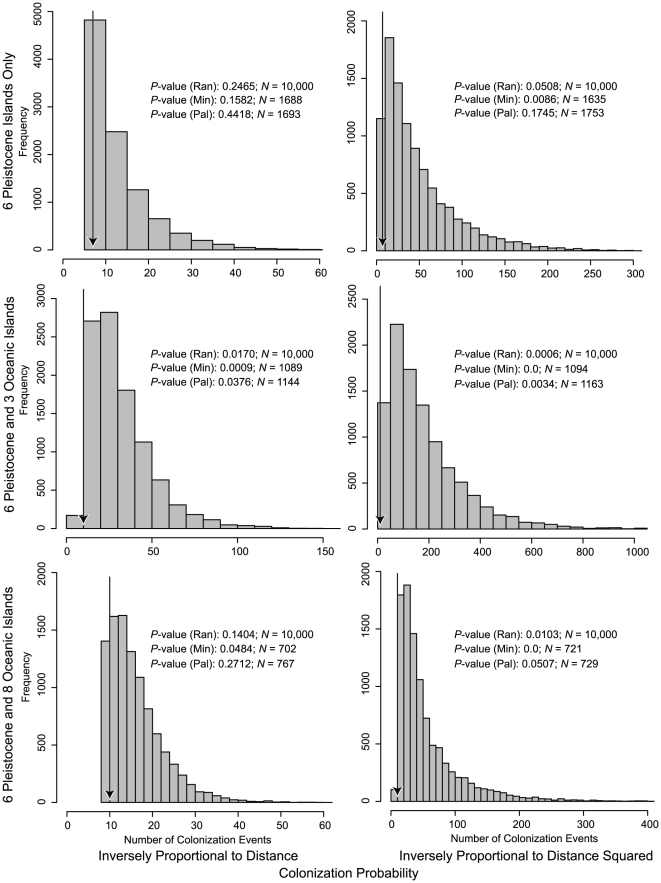
Results of inter-island colonization simulations. Histograms showing numbers of inter-island colonization events necessary to reach a particular number of islands, given a stochastic model of colonization and random starting island are shown. Vertical arrows indicate the minimum number of colonization events necessary to generate populations on 8 of 14 islands, 8 of 9 islands, and 5 of 6 islands, in each case with three islands holding two non-sister species and all others holding one species. *P*-values indicate the proportion of simulations with the number of colonization events less than or equal to the empirical minimum. Scales on x- and y-axes are not equal. *P*-values and sample sizes (*N*) are shown for the entire data set with random starting island (Ran), and for the subsets of replicates that started from near the continental shelf (Mindanao [Min] and Palawan [Pal]).

Our simulations of island colonization suggest it is somewhat unlikely that shrews would colonize all of the currently occupied islands with a random colonization process and the minimum necessary number of dispersal events. When the probability of colonization is inversely proportional to inter-shore distance and the starting island is randomly chosen, the average number of colonization events necessary for shrews to reach 8 of 14 islands is 16.52, for 8 of 9 islands it is 32.25, and for 5 of 6 islands it is 13.11 (Fig. 6). When we make long distance colonization more difficult by using the inverse of squared distances as the probability of colonization, the mean number of dispersal events increases dramatically to 56.89, 181.96 and 49.51, respectively (Fig. 6). The minimum number of colonization events necessary for *Crocidura* to reach all of the islands it is known to occur on, with sympatric, non-sister species pairs present on three islands and one species on all other islands, is 10 (excluding colonization of the first island). This small number of colonization events was rare in the two simulation schemes that required colonization of 8 of 9 islands (*P*≤0.017; Fig. 6). In simulations with a termination criterion of 8 of 14 islands colonized, replicates with 10 or fewer colonization events were somewhat common when long distance colonization was probable (*P* = 0.1404), but rare when long-distance colonization was unlikely (*P* = 0.0103; Fig. 6). If we ignore shrew populations on oceanic islands, only considering the six PAICs (five of which are known to have shrew populations), the minimum necessary number of colonization events that can explain this distribution (three PAICs with two species, two PAICs with one species) is seven. Simulation replicates with seven or fewer colonization events were relatively common when the colonization probability was inversely proportional to distance (*P* = 0.246), but rare when long-distance colonization was simulated as more difficult (*P* = 0.051; Fig. 6). By recalculating these *P*-values for the subset of replicates in which the seeded island was one of the two islands most likely to serve as a colonization routes from the continent to the Philippines, we find that fewer colonization events are necessary from Palawan, but more are necessary from Mindanao, relative to random starting points (Fig. 6).

## Discussion

Testing forces that potentially shape patterns of relatedness and community structure requires a combined approach, because individual tests generally do not provide complete resolution among competing hypotheses [13]. However, even our combined strategy provides only mixed evidence of competition's possible role in determining current patterns of diversity of Philippine shrews. Comparisons of ecological niche models for the two well-sampled species support similarity of abiotic ecological niches, leaving open the possibility for competitive interactions if the two species come into contact. However, the validity of extending this conclusion to other species of Philippine *Crocidura* is debatable. Our tests of phylogenetic dispersion were in the direction of overdispersion (i.e., Δ*Patristic* >0), but not statistically significant. Some degree of overdispersion is expected in this situation—tests of alternative phylogenetic topologies have shown that sympatric species of shrews in the Philippines are not sister species [41]. Therefore, because all speciation events in this clade are thought the result of inter-island colonization, some overdispersion is expected, even in the absence of competitive exclusion. Additionally, statistical power for this test is almost certainly limited because only two pairs of sympatric species are included. Our tests of body-size dispersion, which included all three sympatric species pairs, provide some evidence that co-occurring species may be more divergent in body size than expected by chance. The pattern of body-size differences was significant when using the mean, but not significant when we used the median. The latter approach eliminated the effect of the large difference between the two species on Palawan (*C. batakorum* and *C. palawanensis*). If differences in body size among co-occurring species are non-random, this could be due to either character displacement or a body-size filter that prevents some species from colonizing occupied islands. We suspect the latter is more probable, because there is marginal phylogenetic signal in body size, despite the small clade size.

Our simulations of inter-island colonization indicate that under some scenarios (four of six tests with random starting points were statistically significant), it is unlikely that all the islands that currently hold shrew populations could be colonized with the minimum necessary number of inter-island dispersal events. In other words, ecological factors may have played a role in determining which inter-island dispersal events resulted in successful colonization. If competitive exclusion (or some other factor) is not preventing colonization (and subsequent allopatric speciation), we expect to see a different distribution of species richness in the Philippines. In particular, there should be greater variation in species richness among islands, with high species richness in islands in the center of the archipelago. In contrast to this expectation, we see a conspicuously regular pattern, in which all moderately large islands have only one or two species. However, we acknowledge that there are inherent assumptions built into this model (e.g., random source and direction of colonization) and that our decisions regarding which islands should be included directly affect these expectations. For instance, if we have excluded islands with shrew populations from the simulations, or, if any of the islands we think lack shrews actually hold shrew populations, then our simulated estimates of the numbers of colonization events necessary to populate the archipelago are too low. However, if we have excluded islands that truly lack shrew populations, then our simulations over estimate the numbers of colonization events necessary to populate the archipelago.

Given the potential for unknown species to exist on additional islands, we adopted three geographic scopes in our simulations. First, we included all six PAICs (five of which have shrew populations) and eight oceanic islands (three with shrew populations). However, we note that the mammal faunas of the five oceanic islands and one PAIC lacking shrew records are very poorly known [97], and it remains possible that shrew populations exist on some or all of these islands. In the second approach, we included all oceanic islands and all Pleistocene islands with a record of shrews, plus the largest PAIC that lacks a record (Sulu; Fig. 1), with the expectation that all but one of these islands be colonized. This scenario is liberal in that it excludes oceanic islands that probably have not been colonized. However, it is conservative in that we treated PAICs as cohesive units that only need to be colonized once, despite evidence to the contrary. For instance, the populations of *C. beatus* on Samar and Leyte islands are deeply divergent from other populations on Greater Mindanao [41], [66], perhaps indicating that establishing a shrew population on these islands required an additional colonization event, as if it were an oceanic island. If modern islands within Pleistocene islands have been colonized over water, or over unsuitable habitats, then our treating PAICs as cohesive units would lead to underestimation of the numbers of colonization events in simulations. In our final approach, we ignored the existence of oceanic islands, only considering the six PAICs, five of which are known to have shrew populations. By excluding oceanic islands, we hope to bypass most of the uncertainty associated with the distribution of shrews.

In addition, we examined sets of simulations in which the starting island was randomly chosen, or restricted to either Palawan or Mindanao. Palawan and Mindanao are considered the two primary routes through which relatively recently colonizing organisms have invaded the Philippines [56], [99]. They are considered most important in this sense because of their present proximity to the Sunda Shelf. Our simulations suggest range expansion from Palawan (three of six were statistically significant) may require fewer colonization events than when the organisms originate from Mindanao (five of six were statistically significant). This difference is probably caused by the extremely short distances between Greater Mindanao and Greater Luzon, which leads to frequent back and forth colonization between these two islands being favored over colonization of other areas.

If our chosen geographic scopes and colonization probabilities are reasonable, then numerous potential colonization events may have failed after dispersing shrews arrived on islands already occupied by another species. In effect, this would have limited the number of speciation events by preventing the establishment of isolated populations of species. This interpretation assumes that dispersing individuals would not simply interbreed with local populations. Unfortunately, we have no means of assessing whether these species have the capacity to interbreed. If dispersing individuals do interbreed with resident populations, then a genetic signal should be detectable in the form of polyphyly of island populations. However, the foreign genotypes might be extremely rare and detecting them would require extraordinarily dense sampling. The population-level samples used from *C. beatus* and *C. grayi* in a previous analysis [66] showed no signs of introgression, implying a lack of gene flow. However, we doubt that this sampling was sufficient to detect extremely rare inter-species introgression.

Additional anecdotal evidence suggests body size and/or relatedness may play a role determining species' ability to coexist. We note that in two of the three cases of co-occurring *Crocidura* in the Philippines, one member of the sympatric pair is a restricted range species, perhaps endemic to a single mountain. Specifically, on Mindoro Island, *C. mindorus* is only known from near the peak of Mt. Halcon, but *C. grayi* is widespread and common on the island. Both species have been collected at high elevation on Mt. Halcon, suggesting they are truly sympatric on that mountain. Similarly, on Mindanao Island, *C. grandis* is only known from the type locality at high elevation on Mt. Malindang, but *C. beatus* is widespread on the island and known from numerous localities, including areas sampled on Mt. Malindang. In both cases, surveys of neighboring mountains have failed to capture the apparent micro-endemic species [51, 66, 70, 73; Esselstyn, D. S. Balete, L. R. Heaney unpubl. data]. Thus, it appears that *C. mindorus* and *C. grandis* are each restricted to high elevation areas on one mountain, implying that a narrowing of one species' niche may facilitate coexistence. In contrast, on Palawan Island, *C. batakorum* and *C. palawanensis* are both widely distributed [41]. Patristic distances and differences in body size between these two species are greater than those observed in the other pairs of sympatric species, suggesting that magnitude of body-size and/or phylogenetic distances may contribute to the extent to which species co-occur. However, it should be noted that *C. batakorum* is more closely related to *Crocidura* from Sulawesi than to the other Philippine species [41].

While we acknowledge that our results are mixed, with several non-significant tests, we note that all our test statistics lie on the sides of their respective distributions (e.g., positive Δ*Size* and Δ*Patristic*) that suggest ecological interactions do play a role in determining the outcome of inter-island dispersal events. Statistical power in our analyses is certainly limited by both clade size and uncertainty in the fine-scale geographic distribution of Philippine *Crocidura*. We therefore interpret our results as tenuously suggesting Philippine shrews represent a non-adaptive radiation, in which a lack of ecological innovation may have prevented the accumulation of more than 1–2 species per island. Although the distinction between adaptive and non-adaptive radiations is one of degree [100], we suspect that many terrestrial vertebrates that have diversified within the Philippines are closer to the non-adaptive end of the spectrum [e.g., 90], perhaps because speciation is so often allopatric and associated with inter-island colonization. If our supposition is correct, then a general lack of recent ecological innovation [*sensu* 11, 14, 15] may present a greater hindrance to speciation than does the need to cross the numerous ocean channels that ‘isolate’ the many islands of the Philippines. Although our results are not entirely conclusive, they provide a new perspective and a set of testable hypotheses that potentially explain the accumulation of insular diversity, in which inter-island dispersal is common, but successful colonization rare, and a general lack of ecological innovation constrains archipelago-wide diversity.

## Supporting Information

Appendix S1 Voucher specimens measured for condylo-incisive length.
(TXT)
